# A Hybrid Fuzzy-PSO Framework for Multi-Objective Optimization of Stereolithography Process Parameters

**DOI:** 10.3390/mi16111218

**Published:** 2025-10-26

**Authors:** Mohanned M. H. AL-Khafaji, Abdulkader Ali Abdulkader Kadauw, Mustafa Mohammed Abdulrazaq, Hussein M. H. Al-Khafaji, Henning Zeidler

**Affiliations:** 1 Collage of Production Engineering and Metallurgy, University of Technology-Iraq, Baghdad 10066, Iraq; mohanned.m.hussein@uotechnology.edu.iq (M.M.H.A.-K.); mustafa.m.abdulrazaq@uotechnology.edu.iq (M.M.A.); 2 Mechanical and Mechatronic Engineering Department, College of Engineering, Salahadin University-Erbil, Erbil 44001, Iraq; 3 Institute for Machine Elements, Design and Manufacturing, TU Bergakademie Freiberg, 09599 Freiberg, Germany; henning.zeidler@imkf.tu-freiberg.de; 4 Collage of Mechanical Engineering, University of Technology-Iraq, Baghdad 10066, Iraq; hussein.m.hussein@uotechnology.edu.iq

**Keywords:** stereolithography (SLA) 3D printer, fuzzy logic, Particle Swarm Optimization (PSO), Taguchi method, multi-objective optimization

## Abstract

Additive manufacturing is driving a significant change in industry, extending beyond prototyping to the inclusion of printed parts in final designs. Stereolithography (SLA) is a polymerization technique valued for producing highly detailed parts with smooth surface finishes. This study presents a hybrid intelligent framework for modeling and optimizing the SLA 3D printer process’s parameters for Acrylonitrile Butadiene Styrene (ABS) photopolymer parts. The nonlinear relationships between the process’s parameters (Orientation, Lifting Speed, Lifting Distance, Exposure Time) and multiple performance characteristics (ultimate tensile strength, yield strength, modulus of elasticity, Shore D hardness, and surface roughness), which represent complex relationships, were investigated. A Taguchi design of the experiment with an L18 orthogonal array was employed as an efficient experimental design. A novel hybrid fuzzy logic–Particle Swarm Optimization (PSO) algorithm, ARGOS (Adaptive Rule Generation with Optimized Structure), was developed to automatically generate high-accuracy Mamdani-type fuzzy inference systems (FISs) from experimental data. The algorithm starts by customizing Modified Learn From Example (MLFE) to create an initial FIS. Subsequently, the generated FIS is tuned using PSO to develop and enhance predictive accuracy. The ARGOS models provided excellent performances, achieving correlation coefficients (R^2^) exceeding 0.9999 for all five output responses. Once the FISs were tuned, a multi-objective optimization was carried out based on the weighted sum method. This step helped to identify a well-balanced set of parameters that optimizes the key qualities of the printed parts, ensuring that the results are not just mathematically ideal, but also genuinely helpful for real-world manufacturing. The results showed that the proposed hybrid approach is a robust and highly accurate method for the modeling and multi-objective optimization of the SLA 3D process.

## 1. Introduction

The prevalence of additive manufacturing (AM) has increased significantly in the last 30 years. Researchers and practitioners agree that AM has expanded across aerospace, biomedical engineering, and dental manufacturing [1,2]. There are seven types of these technologies: vat polymerization, material extrusion, material jetting, binder jetting, powder-based fusion, direct energy deposition, and sheet lamination [3,4]. Stereolithography (SLA) is a type of vat polymerization which creates parts by solidifying a liquid photopolymer resin, where it enables high-fidelity parts with strict surface and mechanical requirements [5]. This method uses a focused light source for the initial polymerization, which makes it possible to make highly accurate parts [6,7,8]. SLA machines create objects one layer at a time on a build platform that is slowly lowered into a vat of liquid resin. Each new layer is exposed to the light source [9,10,11].

Numerous process parameters have significant impacts on the quality of the fabricated parts; thus, it is very important to choose the proper combinations of parameters to achieve the desired characteristics [12,13]. A number of investigations have focused on how these factors affect mechanical strength. Li and Teng, for example, explored how printing orientation affected the tensile strength and Young’s modulus of an ABS polymer. They found that, as the orientation angle decreases, the tensile strength increases [14]. Salih et al. concentrated on improving the mechanical characteristics of the polymer resin utilized in the production of a new additive manufacturing composite material including carbon fibers. Their findings indicated that the ultimate strength increased with the addition of carbon fiber to a certain amount; nevertheless, beyond a specific percentage of carbon fiber, the strength diminished [15]. Other investigations have also shown that the anisotropic mechanical characteristics change depending on the orientation of the printed object [16,17,18,19]. Chen et al. [20] investigated how the parameters of irradiation affect the curing and interfacial tensile strength of hydroxyapatite (HAP) pieces made with SLA 3D printing.

Statistical approaches like the Taguchi method and Analysis of Variance (ANOVA) are beneficial for figuring out which process factors are important from a limited number of experiments in this complicated parameter domain [21,22,23]. However, while these methods are effective at identifying which components are important, they do not automatically create a predictive model that can guess or interpolate outcomes for combinations of parameters that were not directly examined. This constraint makes it hard to find important factors and then use them to make predictions and improvements throughout all stages of the process.

This is where intelligent models demonstrate their effectiveness. Artificial Neural Networks (ANNs) and other similar methods have shown promise in predictive modeling; however, they often work as “black box” models, which means they do not provide much information about the physics of the process behind them [24,25,26]. Fuzzy logic, which is one of the AI branches, is a strong alternative. It is particularly beneficial for manufacturing processes because it can deal with uncertainty and represent complicated systems using rules that are similar to those used in human reasoning [27]. In SLA optimization, particularly in regulated applications, interpretability supports traceable, auditable decisions, an advantage over black box ANN models [28]. Mamdani-type fuzzy systems have greater advantages than more opaque models since they are easier to understand [29]. Automated techniques that directly create the fuzzy inference system (FIS) from experimental data, exemplified by the Learning From Example (LFE) algorithms, provide objective and efficient solving [30,31]. Combining these models with optimization methods like Particle Swarm Optimization (PSO) can also make them much better at performing predictions [32,33].

The present study intends to create a strong and clear modeling framework for improving SLA printing. The proposed method uses a multi-stage approach, starting with an effective experimental design that uses the Taguchi method. After that, a new algorithm called ARGOS (Adaptive Rule Generation with Optimized Structure) is developed to automate the process of creating a Mamdani-type FIS from experimental data. The first FIS model is made using a specially designed Modified Learn From Example (MLFE) method, which is refined afterwards by utilizing Particle Swarm Optimization (PSO), overcoming (i) Taguchi’s lack of predictive generalization, (ii) ANN’s black box opacity, and (iii) GA-fuzzy systems’ higher computational cost. “Best part quality” is quantified using a weighted sum objective over tensile and yield strengths, Young’s modulus, Shore D hardness, and surface roughness. This hybrid approach, named ARGOS, makes use of both procedures. In the last stage, a set of tuned fuzzy models is combined with a multi-objective optimization framework to find the best process parameters that give the best part quality. The goal of this study is to show how effectively this hybrid intelligent technique works at developing very accurate predictive models and solving the problem of multi-objective optimization in the SLA printing of ABS.

## 2. Materials and Methods

### 2.1. Materials and Specimen Preparation

**Figure 1:**
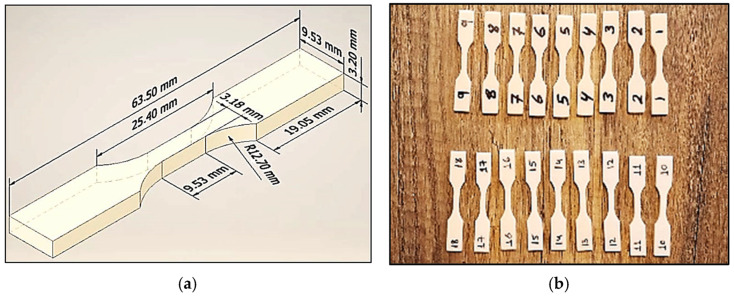
Tensile test specimens; (**a**) dimension of specimen; (**b**) the printed samples.

The specimens for this study were fabricated from ABS-like photopolymer resin using an LD006 SLA 3D printing machine. The printer features a 4K monochrome screen and a build volume of 192 × 120 × 250 mm, and allows for precise control over process parameters. Based on ASTM D638 Type-V, the tensile test specimens were designed using SolidWorks 2024, with dimensions clarified in Figure 1a. The CAD models were exported in STL format and processed using slicing software to generate the G-code for the printer. The process parameters and their respective levels investigated in this study are detailed in Table 1. The final printed samples are shown in Figure 1b.

Parameter ranges (*L_d_* 4–6 mm, *t_E_* 3–7 s) were determined from pilot experiments and manufacturer recommendations to maintain reliable layer separation and curing for the selected resin. Wider ranges, tested preliminarily, introduced delamination (<4 mm) and over-curing (>7 s).

### 2.2. Experimental Design and Testing

To efficiently study the effects of the multiple input parameters on the output responses, a Taguchi L18 orthogonal array was used to design the experiments [21,22]. This statistical method allows for the analysis of a wide parameter space with a reduced number of experimental runs. The L18 array is suitable for accommodating one two-level factor and up to three-level factors, making it a perfect fit for the selected input parameters. The 18 experimental runs prescribed by the array shown in Table 2 formed the basis for the dataset used to train and test the fuzzy logic model.

**Figure 2:**
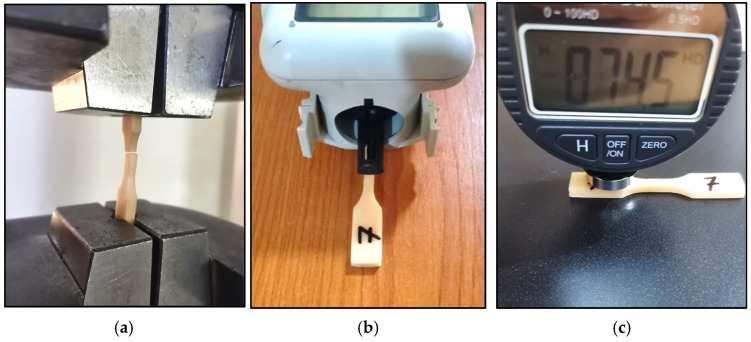
Testing of the samples: (**a**) tensile test, (**b**) surface roughness, (**c**) Shor-D hardness test.

Following fabrication, a series of mechanical tests were performed as depicted in Figure 2. Tensile tests were conducted on a computerized universal testing machine (WDW 200) at a crosshead speed of 1 mm/min to determine ultimate tensile strength ($\sigma_{ult}$), yield strength ($\sigma_{y}$), and Young’s modulus (E). Surface roughness ($R_{a}$) was measured using a Mahr MarSurf-PS1 instrument. Shore D hardness (ShD) was assessed using a digital durometer, providing insights into the material’s resistance to indentation.

### 2.3. Fuzzy Inference System Architecture

The system is built upon a Mamdani-type fuzzy inference system. The rules generated by the ARGOS algorithm take the following form:

$$IF\;O\;is\;A_{i}\;AND\;L_{s}\;is\;B_{i}\;AND\;L_{d}\;is\;C_{i}\;AND\;t_{E}\;is\;D_{i}\;THEN\;Y_{j}\;is\;E_{i}$$

where O is Orientation, $L_{s}$ is Lifting Speed, $L_{d}$ is Lifting Distance, $t_{E}$ is Exposure Time, $Y_{j}$ is the response, $j\left(1\ldots 5\right)$ is the response number, and $A_{i}$, $B_{i}$, $C_{i}$, $D_{i}$, and $E_{i}$ are fuzzy sets defined by Gaussian membership functions. The final crisp output is determined using the Centroid of Area (COA) defuzzification method.

### 2.4. Custom Modified Learn from Example Algorithm Implementation

The custom MLFE function was developed to adapt the standard MLFE algorithm [30,34]. It iteratively builds an initial Mamdani fuzzy rule base from the experimental training data ($D=\left(x^{k},y^{k},k=1\ldots N\right)$, where x is the parameters’ vector and y is the response vector) with several key modifications, detailed in the following algorithm.

**Figure 3:**
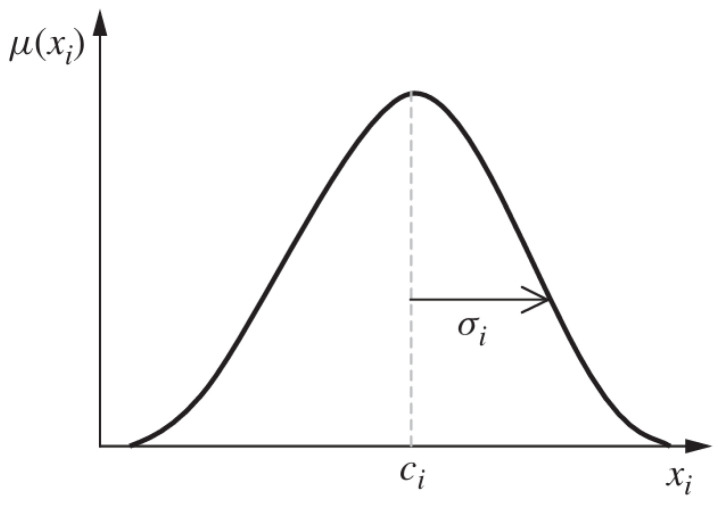
Gaussian membership function.

This algorithm incorporates Gaussian membership functions for inputs $\mu \left(x\right)$.

(1) $$\mu \left(x_{i}\right)=e^{-\frac{1}{2}{\left(\frac{x_{i}-c_{i}}{\sigma_{i}}\right)}^{2}}\;$$

where $x_{i}$ is the ith input variable, $c_{i}$ is the ith center of the membership function (where $\mu \left(x_{i}\right)=1$), and $\sigma_{i}$ is a constant related to the spread of the ith membership function, as shown in Figure 3.

The process is initialized using a starting data point, $\left(x^{s},y^{s}\right)$, where $y^{s}$ is the median of all output values (this is a key feature that differs from [30]), ensuring the initial rule is representative. The starting membership functions and the rule R=1 will be as follows:

(2) $$c_{j}^{1}=x_{j}^{s},\;\;\;\;\;\;\;b^{1}=y^{s},\;and,\;\;\;\;\;\;\;\;\sigma_{j}^{1}=\frac{\max\left(x_{j}\right)-\min\left(x_{j}\right)}{10}$$

(3) $$R=1:IF\;x_{1}\;is\;c_{1}\;AND\;x_{2}\;is\;c_{2}\;AND\ldots .\;AND\;x_{j}\;is\;c_{j}\;THEN\;y_{o}\;is\;b$$

where $c_{j}^{1}$ is the center of the first membership function for the jth input, b is the center of the first membership function of the output, and $\sigma_{j}^{1}$ is the initial spread for the first membership function of the jth input. Additionally, the calculation of $\sigma_{j}^{1}$ is determined by the custom MLFE, which specifies the spread of the initial membership function as 10% of the universe of discourse range. During PSO tuning, $\sigma_{j}^{1}$ was bounded within [5%, 50%] of the range to prevent overly sharp or overly diffuse MFs. Sensitivity tests around the initial $\sigma_{j}^{1}$ showed stable predictions without rule explosion.

This approach represents a distinguishing feature compared to the method described in [30]. For each subsequent data point, a new rule, R, is added only if the prediction error exceeds a predefined tolerance, $\epsilon_{f}$:

(4) $$\left|f\left(x^{k}\right)-y^{k}\right|>\epsilon_{f}\;\;$$

where $f\left(x^{k}\right)$ is the fuzzy logic model output from a given input kth dataset and $y^{k}$ is the experimental response. In this work, $\epsilon_{f}=0.1$. If this condition is met, a new rule, R, is generated. The centers of its input membership function, $c_{j}^{new}$, and output membership function, $b^{new}$, are set directly from the data point:

(5) $$c_{j}^{new}=x_{j}^{k}\;\;\;\;and\;\;\;\;\;\;b^{new}=y^{k}j=1,2,\ldots ,m\;$$

where m is the parameter number. In this work, m=4 (Orientation O, Lifting Speed $L_{s}$, Lifting Distance $L_{d}$, and Exposure Time $t_{E}$). The spread of each new input membership function, $\sigma_{j}^{new}$, is calculated to ensure a smooth interpolation between the new membership function and its nearest existing neighbor. The computation of $\sigma_{j}^{new}$ follows a series of steps. The first step starts with the calculation of the distance between the center of the existing membership functions and the center of the new membership function for each input.

(6) $${\overset{ˇ}{h}}_{j}^{i}=\left\{\left|c_{j}^{i}-c_{j}^{i_{new}}\right|:\;\;i=1,2,\;\ldots ,\;n_{j},\;\;\;i\neq i_{new}\;\;\right\}\;$$

$n_{j}$ is the number of memberships generated for the jth input, and the smallest nonzero distance of the vector ${\overset{ˇ}{h}}_{j}^{i}$, {$\min\left({\overset{ˇ}{h}}_{j}^{i}\right)\neq 0\}$ is the nearest neighbor to the new input membership function. This nearest neighbor’s center, $c_{j}^{i_{min}\;}$, will be used to compute the spread of the new membership function and scale it by a weighting factor, $W_{j}$, as defined in the following equation:

(7) $$\sigma_{j}^{new}=\frac{\left|c_{j}^{i_{new}}-c_{j}^{i_{min}}\right|}{W_{j}}\;$$

A key innovation in custom MLFE is the handling of the “Orientation” parameter. The algorithm ensures that only two membership functions, “Flat” and “Edge”, are ever created, reusing them as needed to prevent redundancy. This enhancement gives the MLFE algorithm the ability to handle input parameters that work with specified states only.

### 2.5. Model Tuning with Particle Swarm Optimization (PSO)

Following the initial generation of FIS by the custom MLFE algorithm, each FIS was subjected to a tuning process to improve its predictive accuracy further. The tuning targets the center of the generated membership functions and their spreads. This optimization was performed in MATLAB 24a using the tunefis function with the Particle Swarm Optimization (PSO) method. PSO is a population-based stochastic optimization technique inspired by the social behavior of bird flocking [35]. It was selected because of its proven efficiency in handling nonlinear, multi-dimensional search spaces and its ability to avoid local minima in fuzzy system tuning. PSO is also computationally less expensive compared to alternatives such as Genetic Algorithms (GAs) or Differential Evolution (DE), making it well-suited for the iterative tuning of fuzzy membership functions.

The algorithm maintains a population, or swarm, of candidate solutions, termed particles. Each particle’s position in the multi-dimensional search space represents a complete set of tunable parameters for the FIS (i.e., the centers and spreads of all Gaussian membership functions).

At each iteration t, the velocity $v_{i}\left(t+1\right)$ and position $x_{i}\left(t+1\right)$ of ith particle are updated according to its own best-known position $(p_{best_{i}}$) and the entire swarm’s best-known position ($g_{best}$), as described in Equations (8) and (9):

(8) $$v_{i}\left(t+1\right)=w\cdot v_{i}\left(t\right)+c_{1}\cdot r_{1}\;\cdot \left(p_{best_{i}}-x_{i}\left(t\right)\right)+c_{2}\cdot r_{2}\cdot \left(g_{best}-x_{i}\left(t\right)\right)$$

(9) $$x_{i}\left(t+1\right)=x_{i}\left(t\right)+v_{i}\left(t+1\right)\;$$

where *w* is the inertia weight, $c_{1}$ and $c_{2}$ are cognitive and social acceleration coefficients, and $r_{1}$ and $r_{2}$ are random numbers between 0 and 1.

This process iteratively guides the swarm towards an optimal set of FIS parameters that minimizes the error between the model’s output and the experimental data. For this study, the membership functions for the “Orientation” input were kept fixed during tuning, as they represent fixed categorical states. PSO hyperparameters: swarm size = 30, max iterations = 200, inertia weight (w = 0.7), acceleration coefficients ($c_{1}$ = $c_{2}$ = 1.5). We executed 10 independent runs with different random seeds and report means. The correlation coefficient was implemented to indicate the accuracy of the generated models. It is calculated as follows [36].

(10) $$R=\frac{n\sum x_{i}y_{i}-\sum x_{i}{\sum y}_{i}}{\sqrt{\left[n\sum x_{i}^{2}-{\left(\sum x_{i}\right)}^{2}\right]\left[n\sum y_{i}^{2}-{\left(\sum y_{i}\right)}^{2}\right]}}$$

The ARGOS algorithm is a hybrid algorithm that combines the custom MLFE and PSO algorithms, and can be summarized in Algorithm 1.
**Algorithm 1.** The Hybrid Algorithm: ARGOS (Adaptive Rule Generation with Optimized Structure)**First.** Input:
D: Training dataset $\left(x^{k},y^{k}\right),k=1\ldots N$.$\epsilon_{f}$: Error tolerance threshold (0.1).W: Weighting factor vector for input spreads (taken 2) for all inputs and output.**Second.** Initialize the Custom Modified From Example algorithm.Create a Mamdani fuzzy inference system FIS object.Initialize the FIS with an empty rule base.Identify starting data point ($x^{s}$,$y^{s}$) where $y^{s}$ is the median of all output values in D.Sort the dataset (x,y) according to the y.Take the median (the points located at the middle).R= 1 (*Initialize Rule counter*).Create First Rule and associate MFs for point ($x^{s}$,$y^{s}$) and initialize $i_{j}=1$ (the membership counter for the jth input) using Equations (2) and (3).Start the loop for each data point $(x^{k},y^{k}$) in D, where k is the dataset loop counter.
Evaluate the FIS with existing rules and membership functions $y_{pred}=f\left(x^{k}\right)$.**if** $|y_{pred}-y^{k}|>\epsilon_{f}$ using Equation (4):
Create new membership functions for each input and output using Equation (5) ($i_{j}=i_{j}+1$).Compute the vector $\overset{ˇ}{h}$ using Equation (6).If ${\overset{ˇ}{h}}_{j}^{i}=0$, it means the new membership of the jth input already exists, and there is no need to generate a new one (the old membership will be used in the New Rule R).Compute the spread for each new membership function using Equation (8).R← R+1.Create New Rule with MFs defined by steps (a to d).If k<N: GOTO 6.If $|y_{pred}-y^{k}|\leq \epsilon_{f}$: stop.
Return FIS.**Third.** Tune the generated FIS using PSO.
Compute the $R^{2}$ using Equation (10).If $R^{2}<0.97$: GOTO Third.Stop.


### 2.6. Multi-Objective Optimization

After the five individual fuzzy models were tuned, a final optimization step was performed to identify a single set of printing parameters that yields the best overall performance. The weighted sum optimization method was employed to combine the multiple objectives into a single objective function (*F*(***x***)) to be minimized:

(11) $$F\left(x\right)=\sum_{i}^{5}w_{i}.f_{i}\left(x\right)\;$$

Weights ($w_{i}$) are assigned as (−1) to properties targeted for maximization (such as strength and hardness), and as (+1) for the property intended to be minimized (roughness). The optimization process was conducted in MATLAB using the “fmincon” function, which is specifically designed for constrained nonlinear minimization. To utilize the minimization capability of the “fmincon” solver for properties that require maximization, the ($w_{i}=-1$) values were adjusted accordingly to transform the maximization objective into a minimization problem.

Each Taguchi L18 condition was tested once due to equipment and material limitations. Although this restricts statistical variability assessment, the findings still provide meaningful trends for process optimization. Future work will include replicate tests to evaluate uncertainty and enhance model robustness.

## 3. Results and Discussion

### 3.1. Taguchi Method Analysis

The results of the measured responses are clarified in Table 2. The full set of engineering stress–strain curves for all tested specimens is provided in the Supplementary Materials for reference. These curves support the consistency of the reported mechanical properties. The Taguchi method was utilized to analyze the influence of each printing parameter on the output responses. Signal-to-Noise (S/N) ratios were calculated for each experimental run. For $\sigma_{ult}$, $\sigma_{y}$, E, and ShD, the “larger-is-better” S/N ratio was used. For surface roughness, the “smaller-is-better” S/N ratio was applied. The mean S/N ratio for each factor level was calculated to determine the relative importance of the parameters.

The rank of each parameter’s effect on the five responses is summarized in Table 3. From the Taguchi analysis, it is evident that Orientation, Lifting Distance, and Exposure Time are the most significant factors affecting all measured properties. Lifting Speed was found to be statistically insignificant across all responses within the tested range. Specimens printed in the edge orientation generally exhibited a higher ultimate tensile strength and Young’s modulus, while the flat Orientation led to better yield stress and surface quality. Exposure Time had a significant positive effect on mechanical responses, though excessive exposure began to show diminishing returns. While replicate builds were not performed due to resource constraints, we included ANOVA F-values and 95% confidence intervals for factor effects to quantify variability. We note the lack of replicates as a limitation and address robustness via cross-validation. Orientation exhibited a statistically significant effect on ***σ_ult_*** and E, which had (*p*-value < 0.05) based on ANOVA results (provided in Supplementary Table S1; edge orientation showed higher means with medium effect sizes).

### 3.2. Fuzzy Logic Model Performance

**Figure 4:**
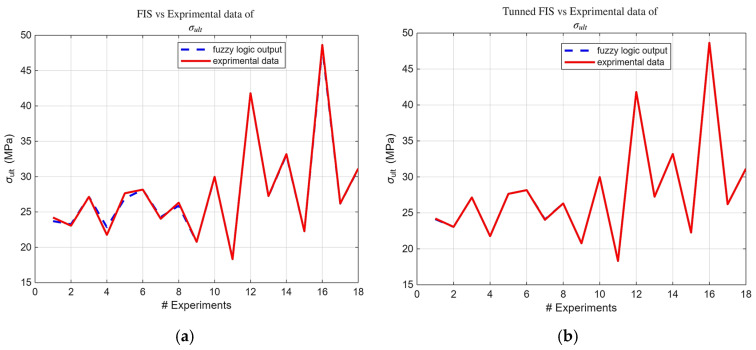
FIS output vs. experimental σ_ult_ data; (**a**) before tuning; (**b**)after tuning.

**Figure 5:**
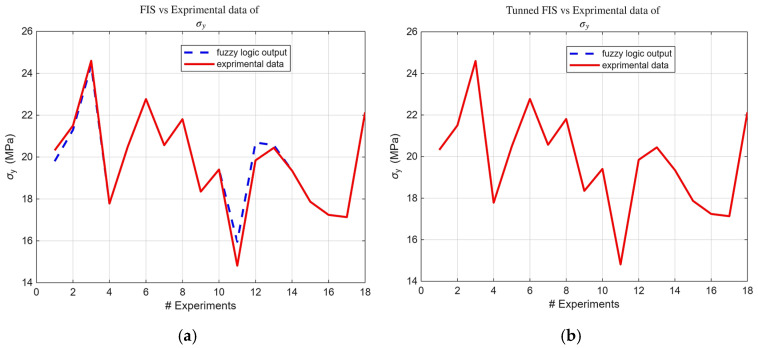
FIS output vs. experimental σ_y_ data; (**a**) before tuning; (**b**) after tuning.

**Figure 6:**
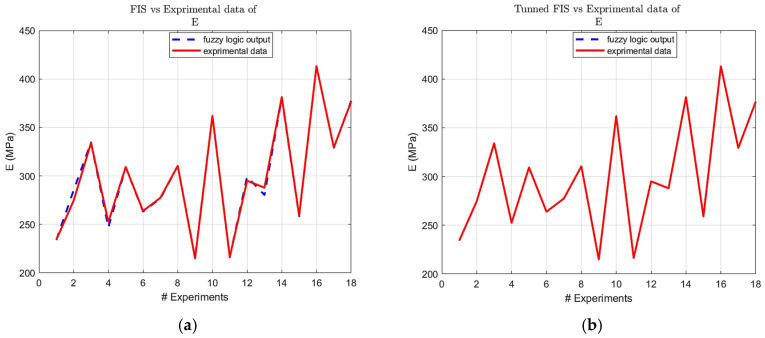
FIS output vs. experimental E data; (**a**) before tuning; (**b**) after tuning.

**Figure 7:**
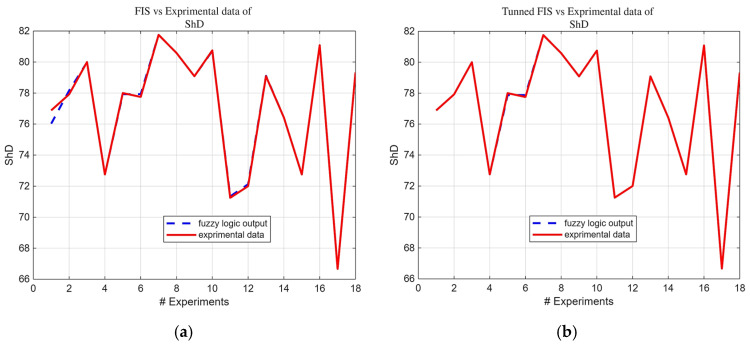
FIS output vs. experimental ShD data; (**a**) before tuning; (**b**) after tuning.

**Figure 8:**
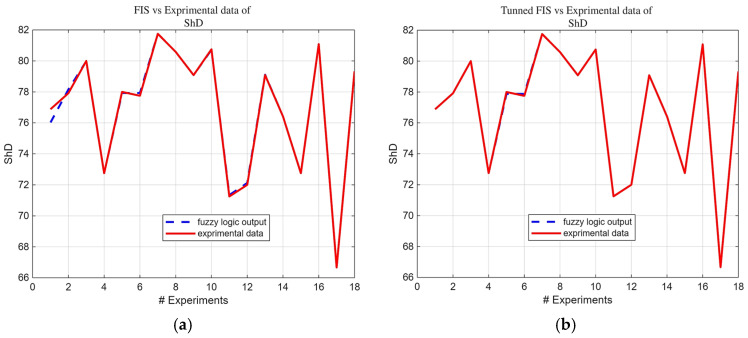
FIS output vs. experimental Ra data; (**a**) before tuning; (**b**) after tuning.

**Figure 9:**
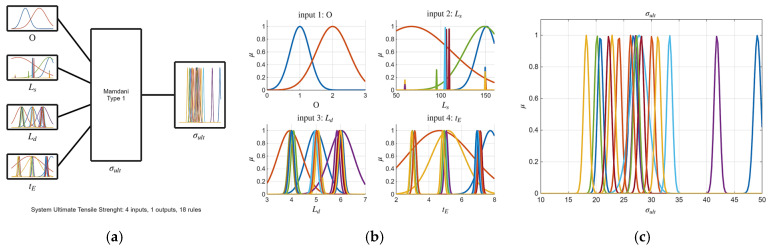
The tuned FIS for σ_ult_ response; (**a**) FIS structure; (**b**) inputs membership; (**c**) output membership functions.

**Figure 10:**
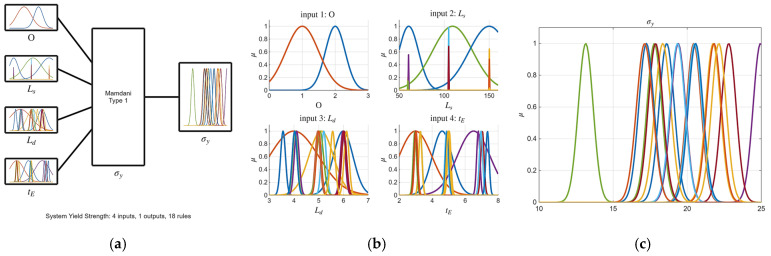
The tuned FIS for σ_y_ response; (**a**) FIS structure; (**b**) inputs membership; (**c**) output membership functions.

**Figure 11:**
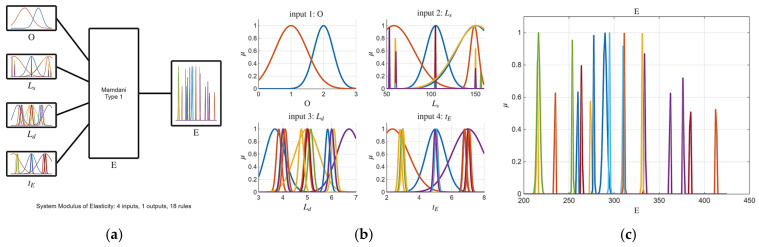
The tuned FIS for E response.; (**a**) FIS structure; (**b**) inputs membership; (**c**) output membership functions.

**Figure 12:**
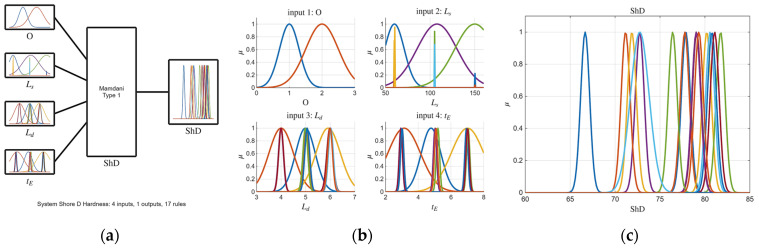
The tuned FIS for ShD response; (**a**) FIS structure; (**b**) inputs membership; (**c**) output membership functions.

**Figure 13:**
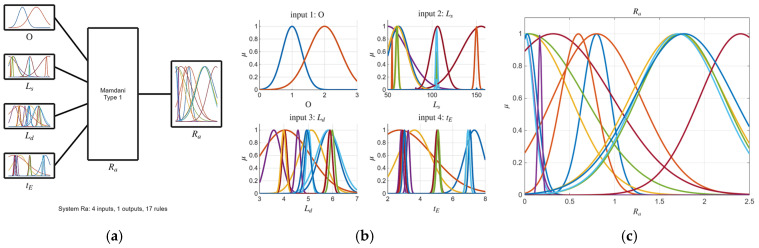
The tuned FIS for Ra response; (**a**) FIS structure; (**b**) inputs membership; (**c**) output membership functions.

Building upon the initial statistical findings, fuzzy logic models were subsequently developed. Notably, the ARGOS algorithm demonstrated a marked improvement, transitioning from the baseline custom MLFE-generated model to the optimally tuned models achieved through Particle Swarm Optimization (PSO). As summarized in Table 4, the preliminary models already exhibited strong correlations ($R^{2}>\;0.97$), but after tuning all models achieved an $R^{2}$ of 0.9999, reflecting a near-perfect agreement with experimental results. Figure 4, Figure 5, Figure 6, Figure 7 and Figure 8 provide a visual comparison of model predictions and experimental measurements, highlighting the high fidelity of the tuned fuzzy inference systems (FISs). Additionally, Figure 9, Figure 10, Figure 11, Figure 12 and Figure 13 illustrate the architecture of the generated FISs, detailing their inputs, outputs, and rule bases. Overall, these results underscore the ARGOS algorithm’s capacity to deliver highly accurate and reliable predictions, enhancing both the precision and confidence in the modeling process.

The comparison plots (Figure 4, Figure 5, Figure 6, Figure 7 and Figure 8) were generated directly from MATLAB using the FIS evaluation module. Although residual plots were not added due to MATLAB code structure constraints, the linear and unbiased correlation between experimental and predicted values demonstrates a consistent model accuracy across all responses.

The final step in this work is the implementation of the multi-objective optimization. The optimized parameters for the SLA 3D printer are given in Table 5. Also, the optimized printed part properties are presented in Table 6.

### 3.3. Experimental Validation of the ARGOS Framework

To further verify the generalization capability of the developed ARGOS models, five additional experiments were conducted beyond the Taguchi L18 design. Four validation samples were fabricated using new parameter combinations within the studied ranges, and one sample (Exp. 1) was printed using the optimal parameters obtained from the multi-objective optimization results. The experimentally measured responses are illustrated in Table 7, while Table 8 presents the corresponding comparison of measured versus ARGOS predicted values. As shown, the validation results achieved strong correlation coefficients (R^2^ = 0.911 for ultimate tensile strength, 0.933 for yield strength, 0.841 for Young’s modulus, 0.885 for Shore D hardness, and 0.911 for surface roughness).

**Figure 14:**
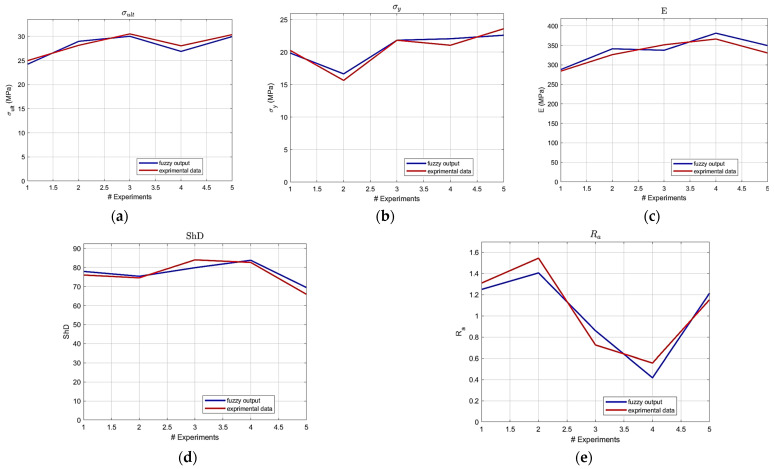
Experimental values vs. prediction outcomes: (**a**) ultimate tensile strength, (**b**) yield strength, (**c**) Young’s modulus, (**d**) Shore D hardness, (**e**) surface roughness.

These results confirm that the hybrid MLFE–PSO framework exhibits a consistent predictive performance even for unseen process conditions, validating the robustness of the proposed modeling approach. The representative comparison plots between experimental and predicted values for the validation samples are illustrated in Figure 14a–e.

## 4. Conclusions

This research successfully developed and validated a hybrid intelligent system for modeling and optimizing the SLA process for ABS photopolymer material. The study demonstrated that the proposed ARGOS algorithm, which hybridized the custom MLFE with PSO, can create exceptionally accurate predictive models. ARGOS achieved very high fold-averaged R^2^ values (>0.99) under a cross-validation of the L18 dataset. While promising, these results should be validated on larger datasets and materials to rule out inadvertent overfitting, surpassing accuracies typically reported for traditional methods like RSM and standard ANNs [25,26,37]. This corroborates findings that hybridizing fuzzy logic with evolutionary algorithms yields superior results [32]. The final multi-objective optimization provides a tangible, actionable set of optimal parameters, reducing the need for costly trial-and-error. Future work could apply the ARGOS framework to other materials or may serve as an alternative to the multi-objective techniques like Non-dominated Sorting Genetic Algorithm NSGA-II in scenarios prioritizing interpretability and low computational overhead [38]. Ultimately, this work contributes a robust, transparent, and highly accurate framework for the intelligent optimization of the SLA process.

## Figures and Tables

**Table 1:** Process parameters and levels.

No.	Parameter	Symbol	Level			Unit
			(1)	(2)	(3)	
1	Build Orientation	*O*	Flat (0°)	On edge (90°)	---	degree
2	Lifting Speed	*L* *_s_*	60	105	150	mm/h
3	Lifting Distance	*L* *_d_*	4	5	6	mm
4	Exposure Time	*t_E_*	3	5	7	s

**Table 2:** Parameter combinations for the L18 Taguchi design.

No.	*O* (θ)	*L_S_* (mm/h)	*L_d_* (mm)	*t_E_* (s)	*σ_ult_* (MPa)	*σ_y_* (MPa)	*E* (MPa)	*Ra* (μm)	*ShD*
1	Flat	60	4	3	24.21	20.32	234.22	0.71	76.88
2	Flat	60	5	5	23.06	21.50	274.63	0.08	77.92
3	Flat	60	6	7	27.15	24.60	334.05	0.80	80.00
4	Flat	105	4	3	21.76	17.78	252.50	0.15	72.75
5	Flat	105	5	5	27.65	20.49	309.20	0.17	78.00
6	Flat	105	6	7	28.17	22.77	263.94	0.09	77.75
7	Flat	150	4	5	24.04	20.57	277.47	0.66	81.75
8	Flat	150	5	7	26.32	21.81	310.44	0.10	80.58
9	Flat	150	6	3	20.77	18.35	214.92	0.60	79.08
10	Edge	60	4	7	29.99	19.41	361.96	1.63	80.75
11	Edge	60	5	3	18.32	14.81	216.51	0.87	71.25
12	Edge	60	6	5	41.81	19.84	295.04	1.64	72.00
13	Edge	105	4	5	27.26	20.44	287.92	1.27	79.08
14	Edge	105	5	7	33.19	19.35	381.42	1.54	76.42
15	Edge	105	6	3	22.27	17.87	259.10	2.04	72.75
16	Edge	150	4	7	48.64	17.24	413.07	1.15	81.08
17	Edge	150	5	3	26.21	17.13	329.34	1.67	66.67
18	Edge	150	6	5	31.15	22.14	376.78	1.03	79.33

**Table 3:** S/N ratio response ranks for process parameters.

	*σ_ult_*	*σ_y_*	*E*	*Ra*	*ShD*
***O***	1	1	1	1	2
***L_S_***	4	4	4	4	3
***L_d_***	2	2	2	3	4
***t_E_***	3	3	3	2	1

**Table 4:** Comparison of model performance ($R^{2}$) before and after tuning.

Response	Before Tuning	After Tuning
***σ_ult_***	0.9976	0.9999
***σ_y_***	0.9786	0.9999
***E***	0.9934	0.9999
***ShD***	0.9880	0.9999
***Ra***	0.9852	0.9999

**Table 5:** Optimized SLA 3D printer parameters.

Optimized Factor	Value	Description
***O***	1	Edge for 1, Flat for 2
***L_S_***	100.33	mm/h
***L_d_***	4.9862	mm
***t_E_***	5	s

**Table 6:** The optimized 3D printed part properties.

Optimized Factor	Value	Description
***σult***	24.223	MPa
***σy***	19.849	MPa
***E***	287.84	MPa
***ShD***	77.941	
***Ra***	1.25	μm

**Table 7:** Validation experiments.

No.	*O* (θ)	*L_S_* (mm/h)	*L_d_* (mm)	*t_E_* (s)	*σ_ult_* (MPa)	*σ_y_* (MPa)	*E* (MPa)	*ShD*	*Ra* (μm)
1	Edge	100	5	5	24.97	20.25	283.89	76.042	1.31
2	Edge	130	5	7	28.15	15.65	326.24	74.58	1.545
3	Flat	70	4	7	30.52	21.82	351.45	84	0.725
4	Flat	130	4	7	28.05	21.05	366.28	82.66	0.556
5	Edge	70	6	3	30.37	23.59	330.47	65.91	1.152

**Table 8:** Experimental values vs. prediction outcomes.

No.	Exp. *σ_ult_* (MPa)	Pred. * σ_ult_* (MPa)	Exp. * σ_y_* (MPa)	Pred. * σ_y_* (MPa)	Exp. * E* (MPa)	Pred. * E* (MPa)	Exp. * ShD *	Pred. * ShD *	Exp. * Ra* (μm)	Pred. * Ra* (μm)
1	24.97	24.22	20.25	19.849	283.89	287.84	76.042	77.94	1.31	1.25
2	28.15	28.98	15.65	16.65	326.24	341.24	74.58	75.38	1.545	1.40
3	30.52	30.03	21.82	21.82	351.45	337.45	84	79.89	0.725	0.85
4	28.05	26.89	21.05	22.05	366.28	381.28	82.66	83.78	0.556	0.42
5	30.37	29.97	23.59	22.59	330.47	349.47	65.91	69.46	1.152	1.21

## Data Availability

The data presented in this study are available on request from the corresponding author due to copyright reasons.

## Supplementary Materials

The following supporting information can be downloaded at https://www.mdpi.com/article/10.3390/mi16111218/s1. Figure S1: Stress-strain curves for the 18 specimens; Tables S1–S5: ANOVA results for the ultimate tensile strength, yield strength, Young’s modulus, shore D hardness, and, surface roughness, respectively; and Figure S2: Stress-strain curves for the validation experiments.
