# Supplementary material for: A Hybrid Fuzzy-PSO Framework for Multi-Objective Optimization of Stereolithography Process Parameters
Source: Micromachines (Basel). 2025 Oct 26;16(11):1218. doi: 10.3390/mi16111218 (PMC12654128; doi:10.3390/mi16111218)
Supplement: Supplementary file 1 [file micromachines-16-01218-s001.zip › micromachines-3900384-supplementary.pdf]

Supplementary Materials

Stress-Strain Curves

The stress-strain curves for the 18 samples are shown in Figures S1 (a) to (r).

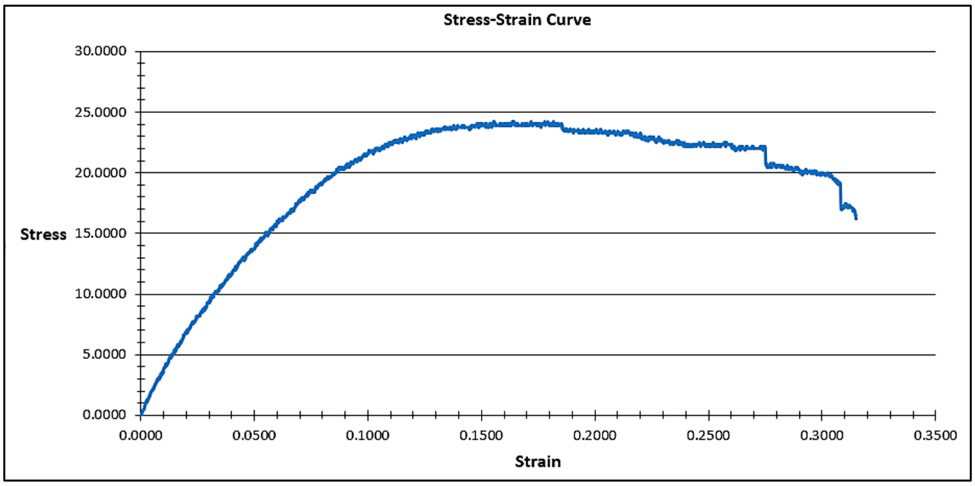

(a) Specimen 1.

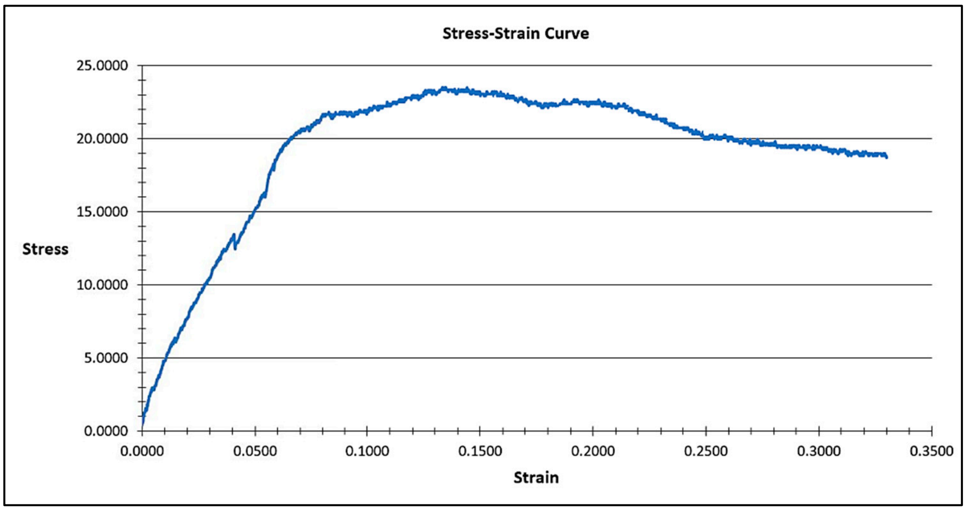

(b) Specimen 2.

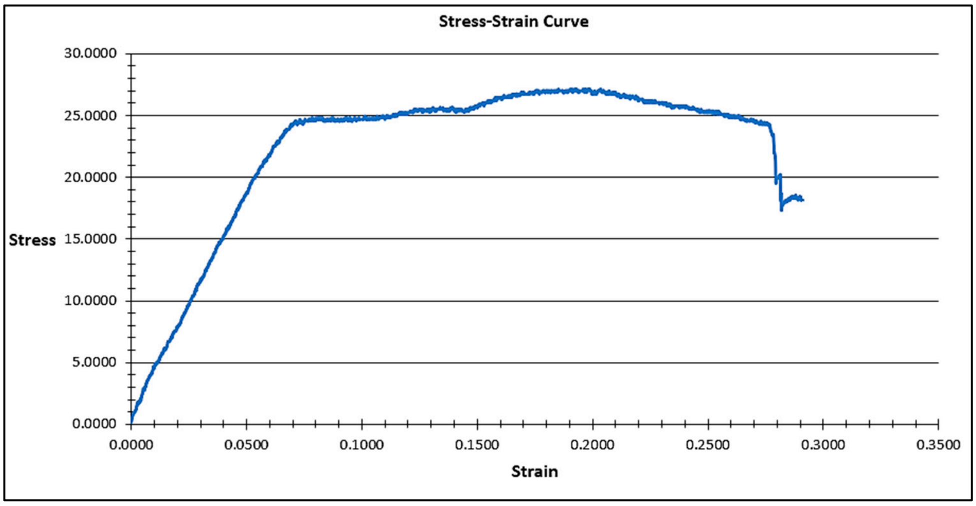

(c) Specimen 3.

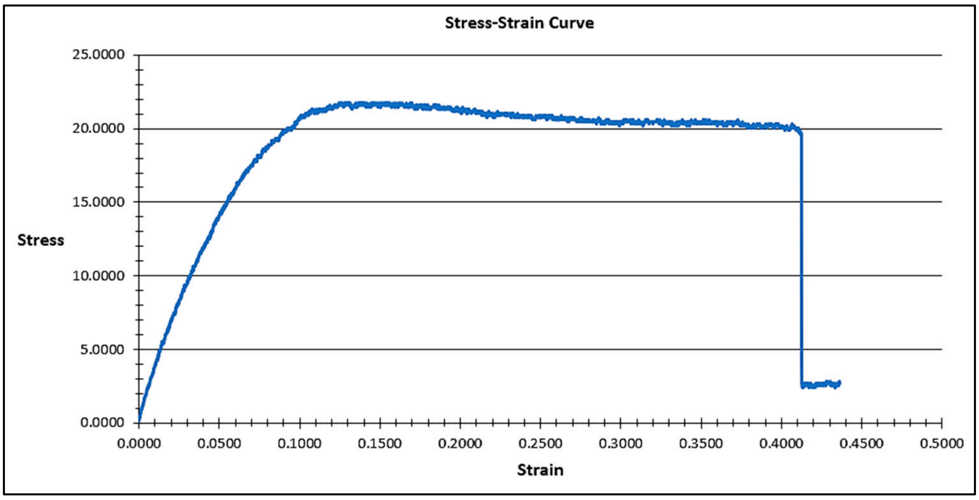

(d) Specimen 4.

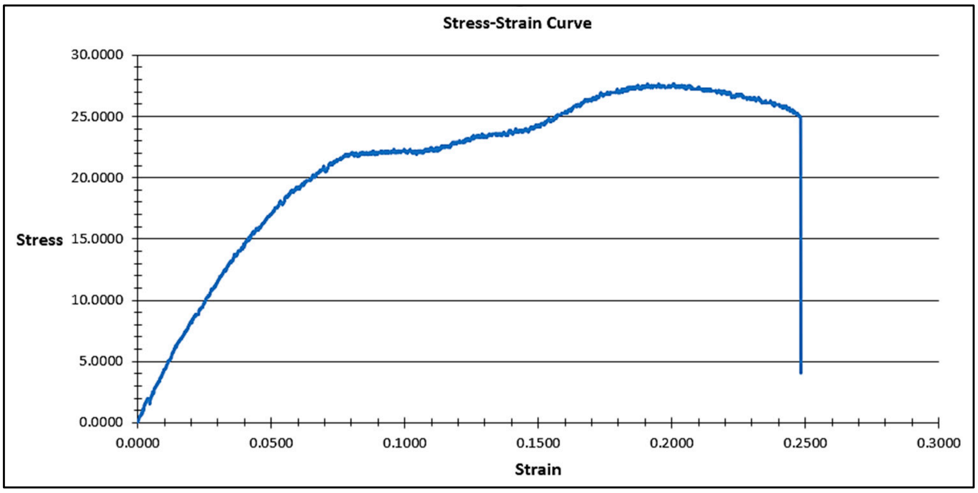

(e) Specimen 5.

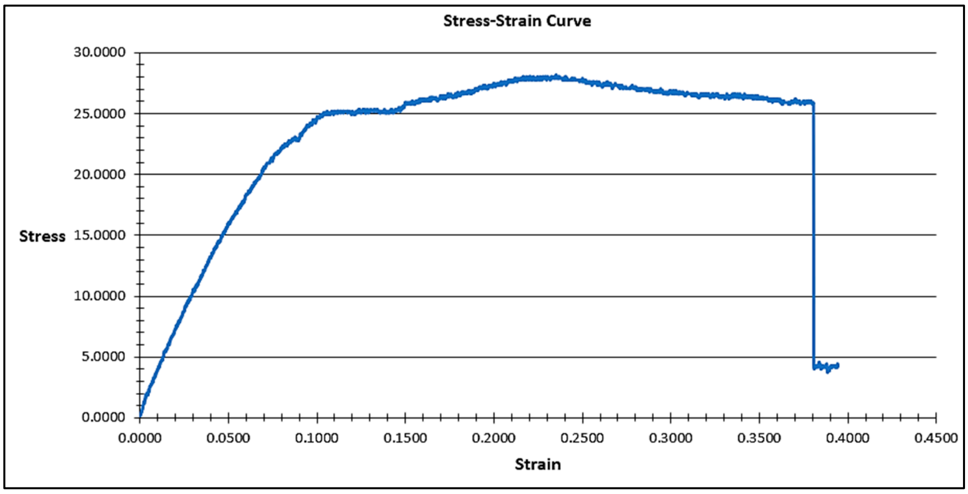

(f) Specimen 6.

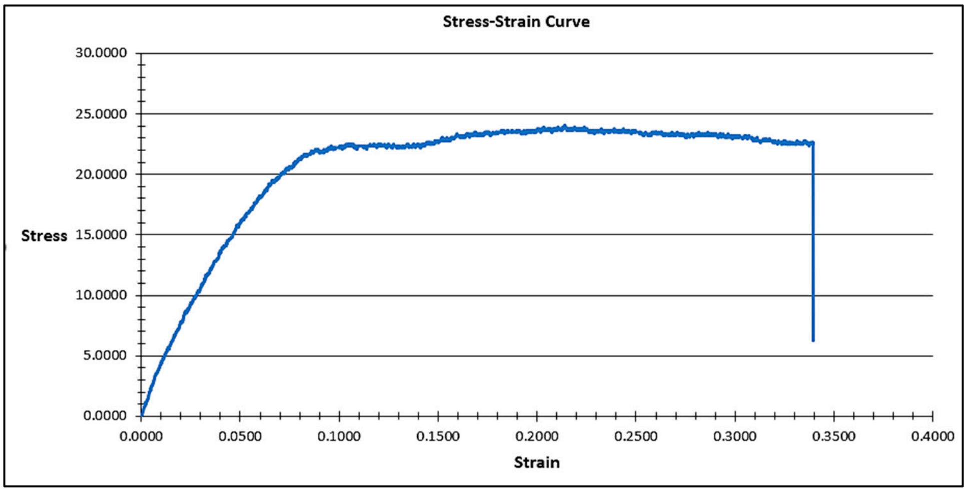

(g) Specimen 7.

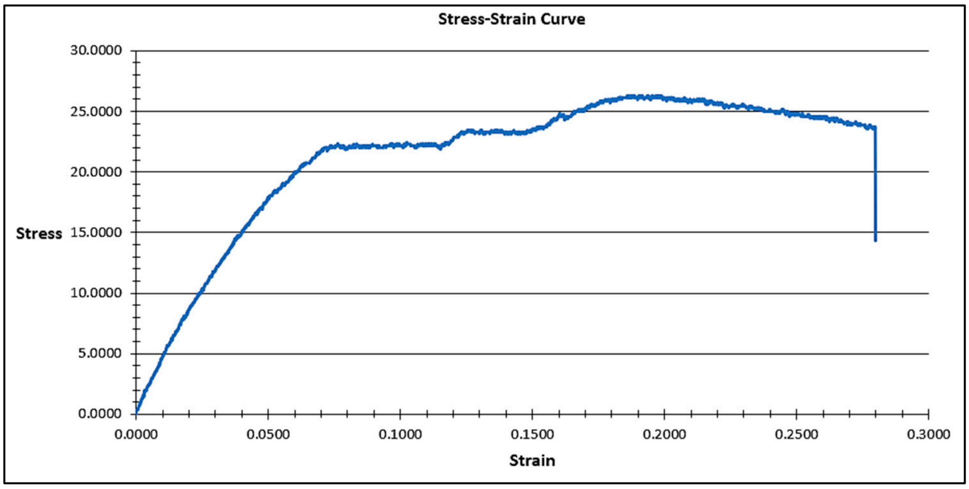

(h) Specimen 8.

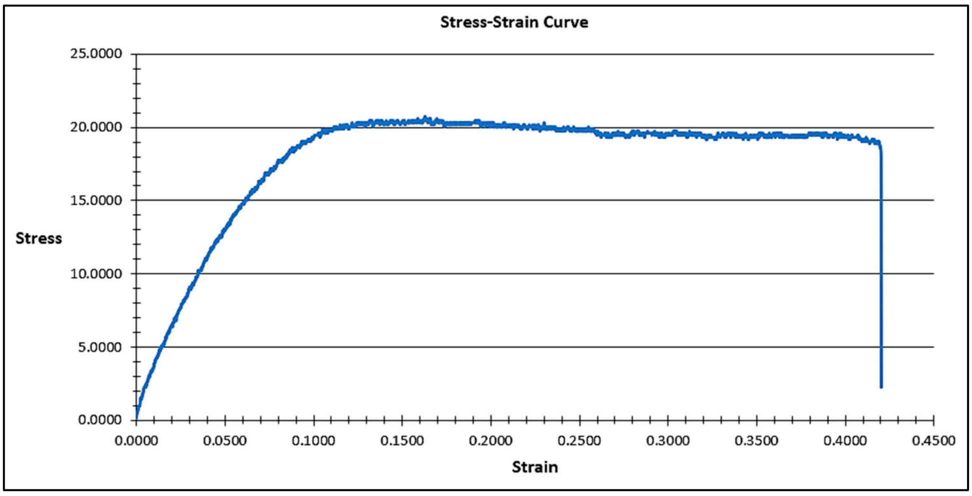

(i) Specimen 9.

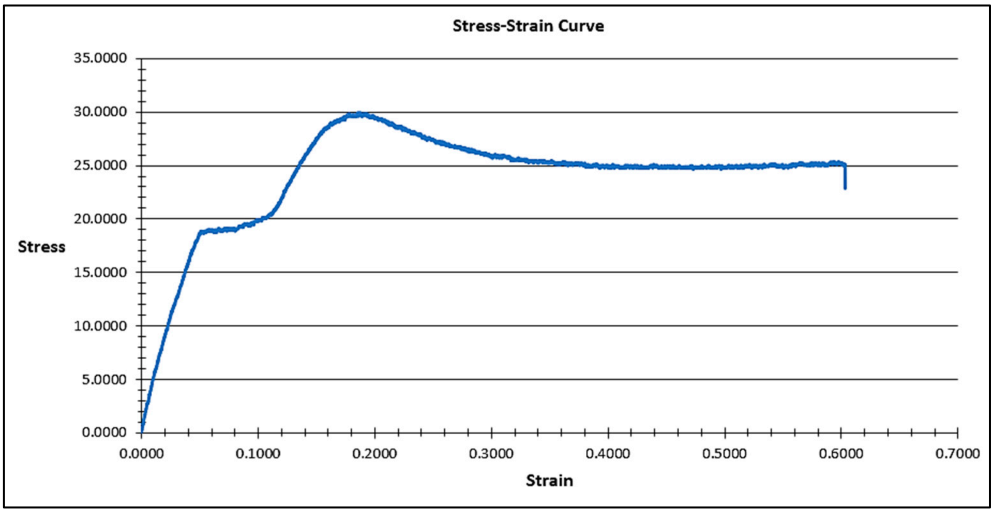

(j) Specimen 10.

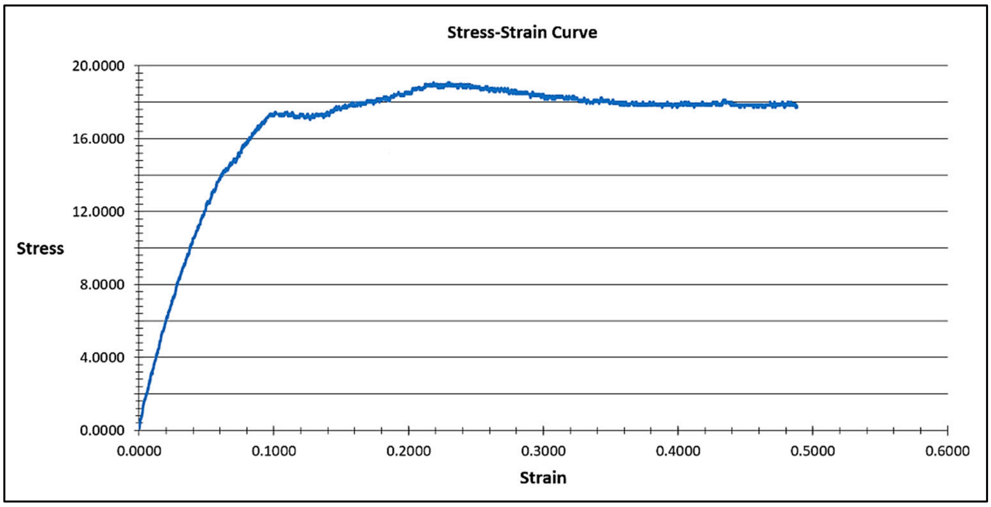

(k) Specimen 11.

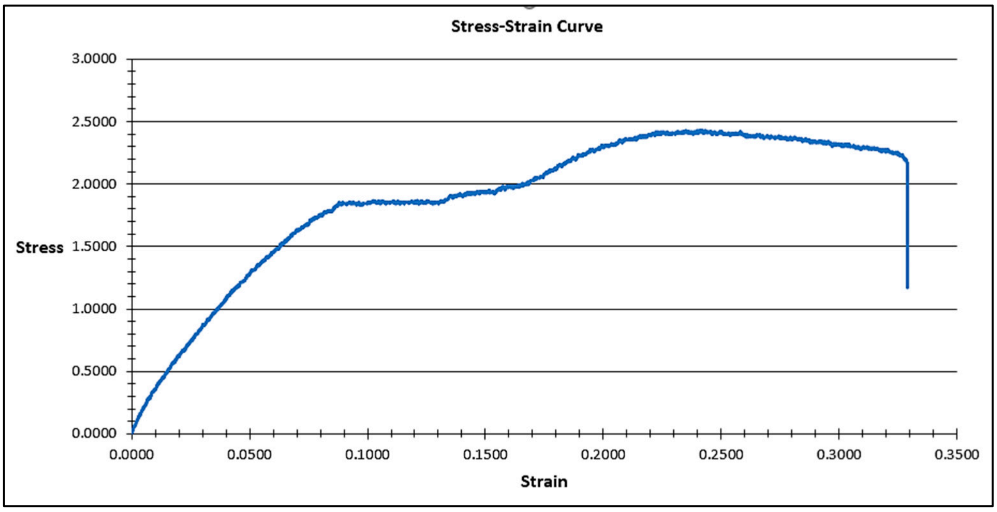

(l) Specimen 12.

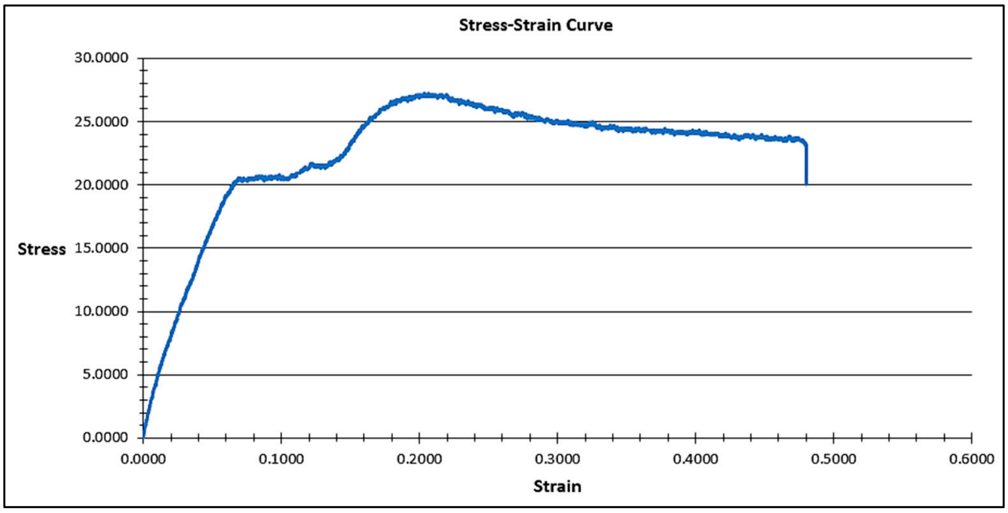

(m)Specimen 13.

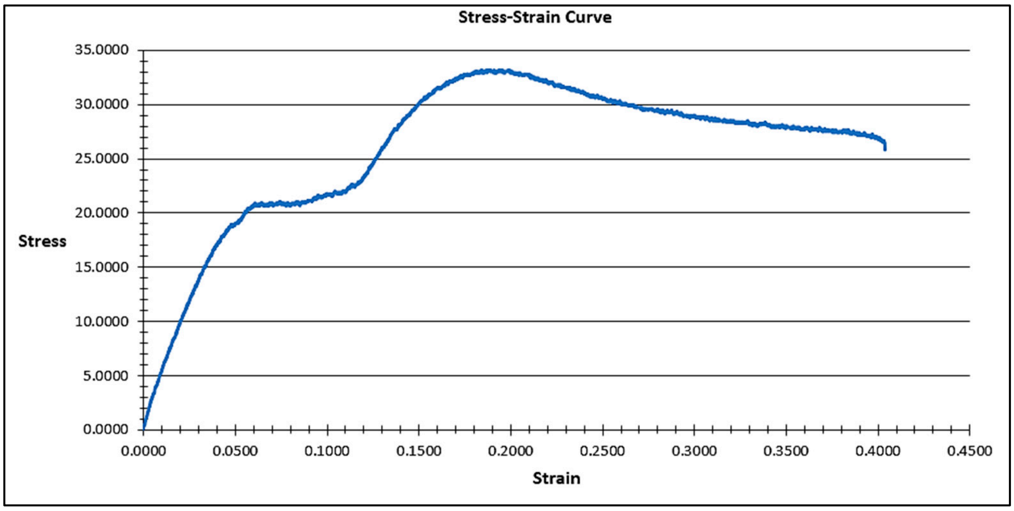

(n) Specimen 14.

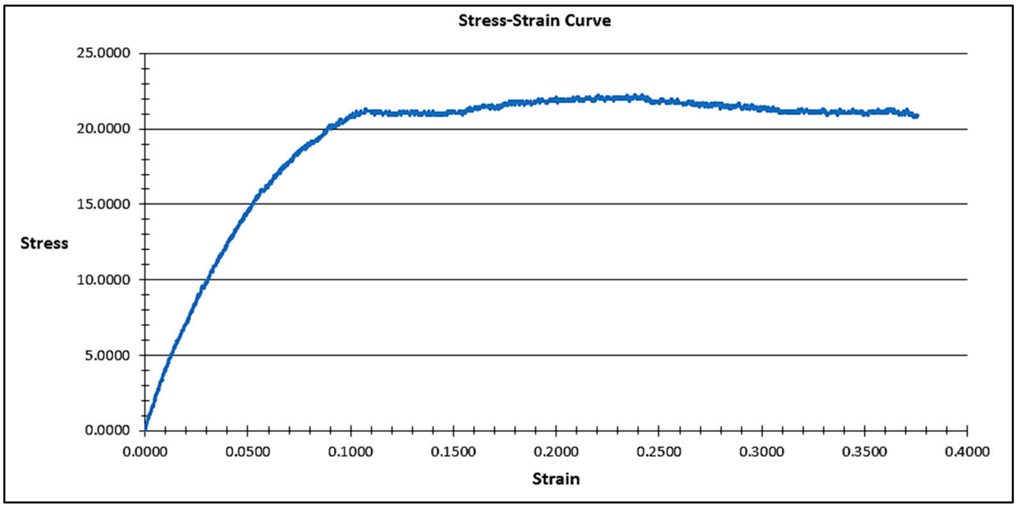

(o) Specimen 15.

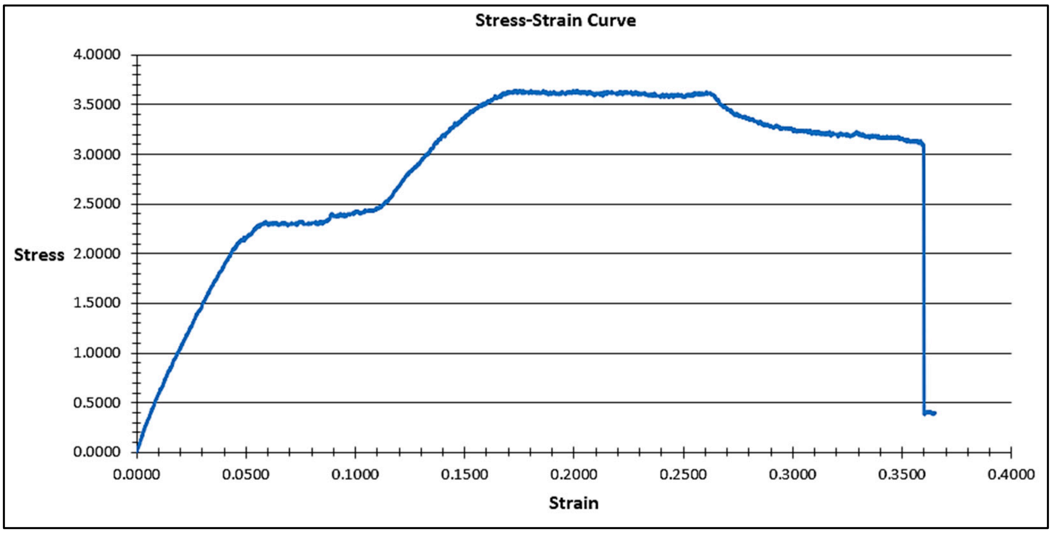

(p) Specimen 16.

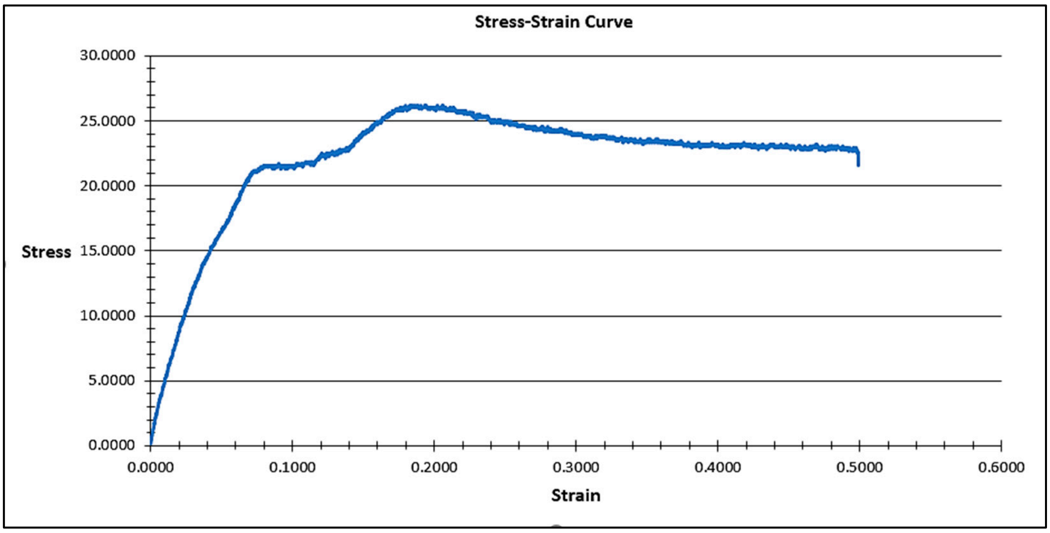

(q) Specimen 17.

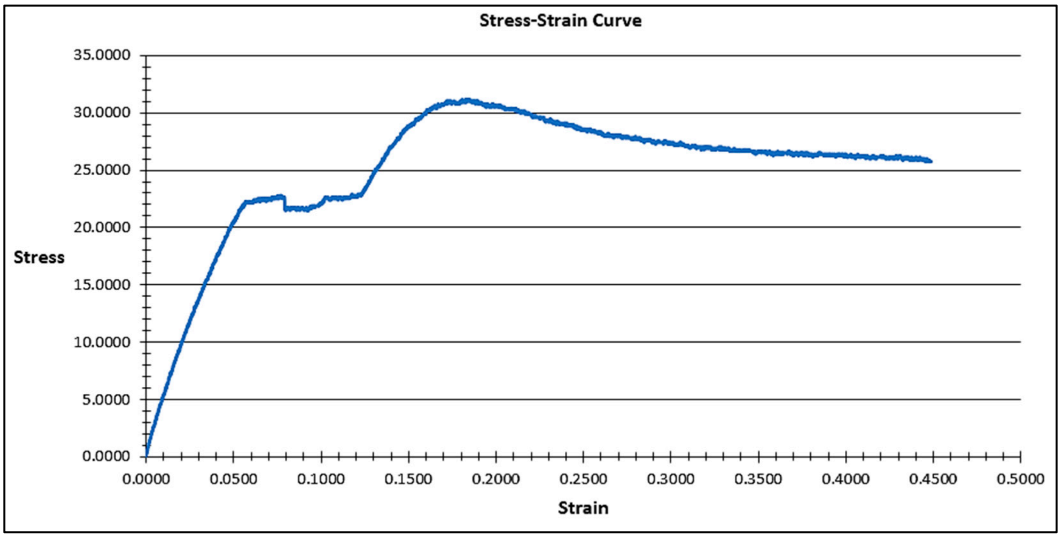

(r) Specimen 18.

Figures S1: (a) to (r) Stress-strain curves for the 18 specimens.

ANOVA Tables

The ANOVA results of the five responses illustrated in Tables S (1) to (5).

Table S1. ANOVA results for Ultimate Tensile Strength.

Source	DF	Adj SS	Adj MS	F-Value	P-Value
Orintation	1	172.38	172.38	4.49	0.049
Lifting speed	2	25.52	12.76	0.33	0.725
Lifting distance	2	41.33	20.67	0.54	0.600
Exposure time	2	313.75	156.87	4.08	0.051
Error	10	384.19	38.42		
Total	17	937.16			

Table S2. ANOVA results for Yield Strength.

Source	DF	Adj SS	Adj MS	F-Value	P-Value
Orintation	1	22.1245	22.1245	10.54	0.009
Lifting speed	2	0.8831	0.4415	0.21	0.814
Lifting distance	2	11.4632	5.7316	2.73	0.113
Exposure time	2	39.3304	19.6652	9.37	0.005
Error	10	20.9949	2.0995		
Total	17	94.7961			

Table S3. ANOVA results for Young's Modulus.

Source	DF	Adj SS	Adj MS	F-Value	P-Value
Orintation	1	11238.6	11238.6	7.82	0.019
Lifting speed	2	3994.9	1997.4	1.39	0.294
Lifting distance	2	722.6	361.3	0.25	0.783
Exposure time	2	26112.4	13056.2	9.08	0.006
Error	10	14378.7	1437.9		
Total	17	56447.2			

Table S4. ANOVA results for Shore D Hardness.

Source	DF	Adj SS	Adj MS	F-Value	P-Value
Orintation	1	35.80	35.795	4.55	0.043
Lifting speed	2	13.13	6.565	0.83	0.462
Lifting distance	2	38.45	19.224	2.44	0.137
Exposure time	2	126.65	63.327	8.04	0.008
Error	10	78.75	7.875		
Total	17	292.77			

Table S5. ANOVA results for Surface Roughness.

Source	DF	Adj SS	Adj MS	F-Value	P-Value
Orintation	1	5.00721	5.00721	34.58	0.000
Lifting speed	2	0.02788	0.01394	0.10	0.909
Lifting distance	2	0.26548	0.13274	0.92	0.431
Exposure time	2	0.11862	0.05931	0.41	0.675
Error	10	1.44791	0.14479		
Total	17	6.86711			

Stress-Strain Curves for the Validation Experiments

The stress-strain curves for the five validation experiments are shown in Figures S2 (a) to (e).

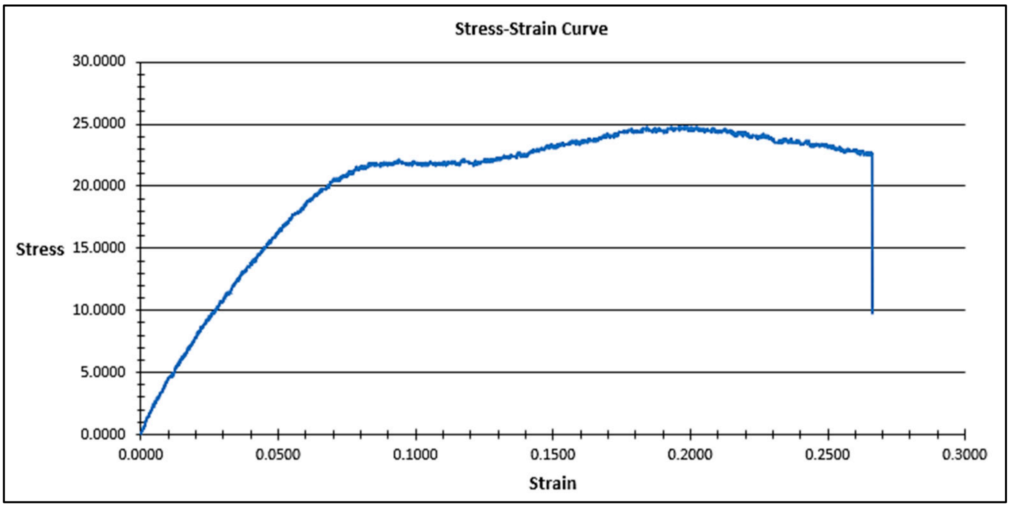

(a) Specimen v1.

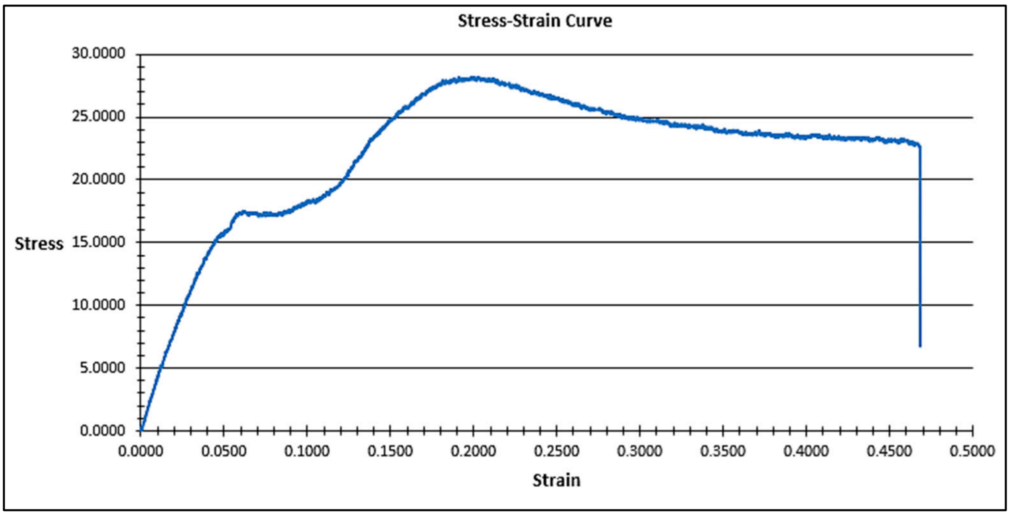

(b) Specimen v2.

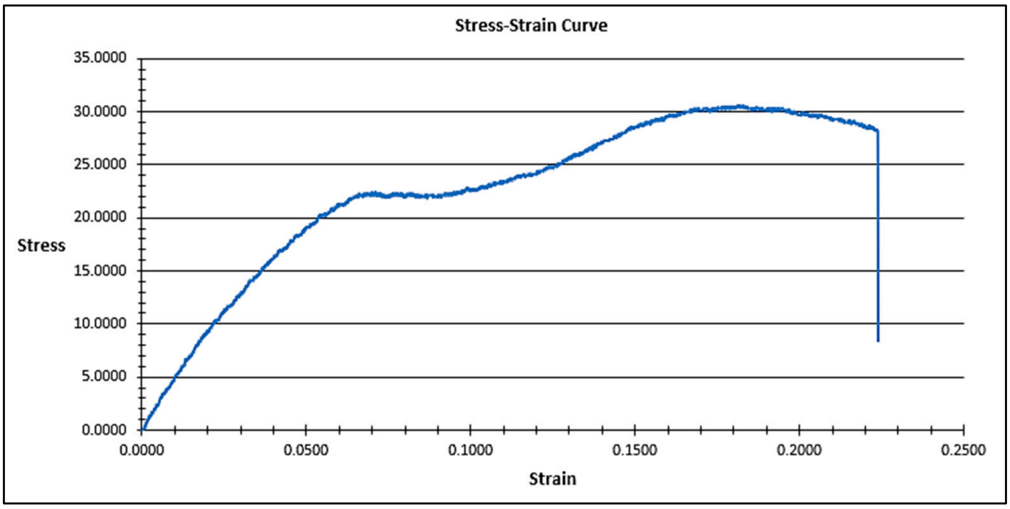

(c) Specimen v3.

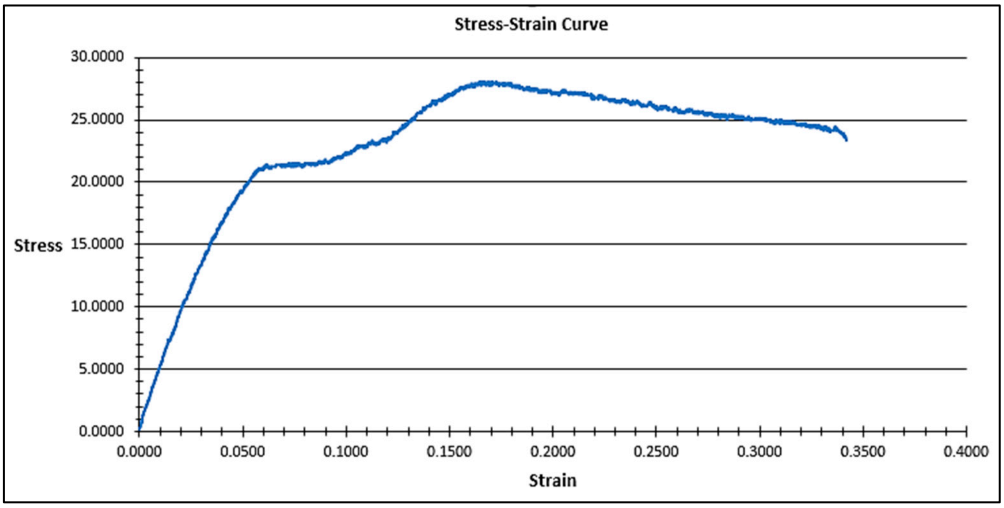

(d) Specimen v4.

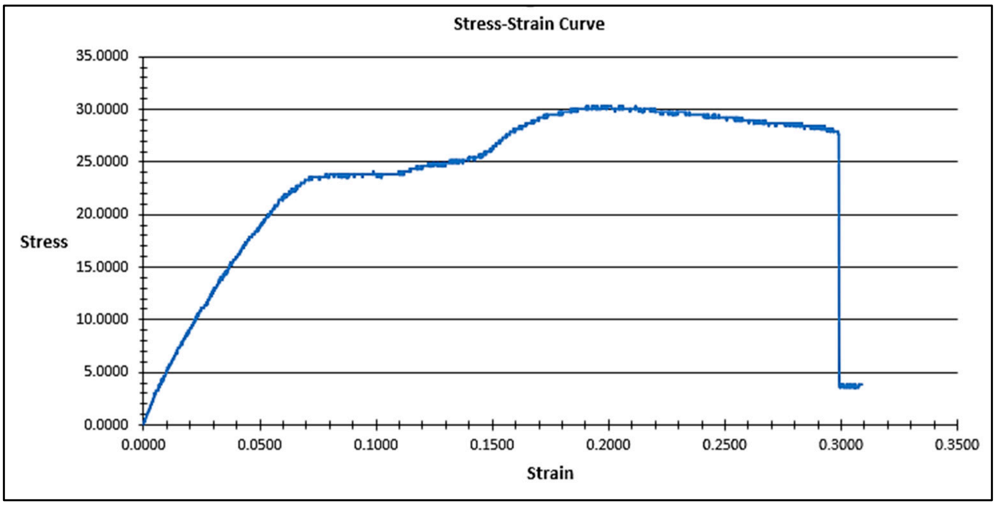

(e) Specimen v5.

Figures S2: (a) to (e) Stress-strain curves for the five validation experiments.
